# Subthreshold Photocoagulation Using Endpoint Management in the PASCAL® System for Diffuse Diabetic Macular Edema

**DOI:** 10.1155/2018/7465794

**Published:** 2018-01-31

**Authors:** Masafumi Hamada, Kishiko Ohkoshi, Keiji Inagaki, Nobuyuki Ebihara, Akira Murakami

**Affiliations:** ^1^Department of Ophthalmology, Juntendo University Graduate School of Medicine, 2-1-1 Hongo, Bunkyo-ku, Tokyo 113-8421, Japan; ^2^Department of Ophthalmology, St. Luke's International Hospital, 9-1 Akashi-cho, Chuo-ku, Tokyo 104-8560, Japan; ^3^Department of Ophthalmology, Juntendo University Urayasu Hospital, 1-1 Tomioka 2-chome, Urayasu-shi, Chiba 279-0021, Japan

## Abstract

We evaluated subthreshold photocoagulation using endpoint management (EPM) for the treatment of diabetic macular edema (DME). The study enrolled 10 eyes from 10 patients (6 men and 4 women) with DME. The entry criteria included central macular thickness (CMT) ≥ 300 *μ*m and decimal visual acuity (VA) ≤ 0.5. The primary endpoints were VA (logMAR) and CMT at 6 months follow-up. Secondary endpoints included fundus autofluorescence, macular volume (MV), and macular sensitivity (MS). We used the PASCAL Streamline Yellow® (wavelength, 577 nm) system to perform grid pattern laser photocoagulation at 50% of the threshold (size, 100 *μ*m; duration, 0.015 s; spacing, 0.5; and energy, 4.5–7.8 mJ). At 6 months posttreatment, CMT was significantly decreased, while there were no significant changes in macular sensitivity, mean BCVA (logMAR), or macular volume. Autofluorescence imaging revealed no changes after treatment in 6 of 10 eyes. No eyes exhibited subjective symptoms of scotoma after photocoagulation. Optical coherence tomography showed the complete resolution of macular edema in 4 eyes (40%) after a single treatment; MS was increased in all 4 of these eyes at 6 months posttreatment. In conclusion, subthreshold photocoagulation using EPM is safe and effective for DME treatment and preserves MS. This trial is registered with UMIN000012401.

## 1. Introduction

Over 200 million people worldwide have diabetes mellitus. Accordingly, diabetic retinopathy is a significant cause of impaired vision in working-age populations in developed countries [1–3]. Visual impairment in diabetic retinopathy is mainly caused by diabetic macular edema (DME), in which leakage from microaneurysms and blood vessels causes abnormal macular structure and photoreceptor damage [4, 5]. In 1985, the Early Treatment Diabetic Retinopathy Study (ETDRS) showed that laser photocoagulation decreased the risk of vision impairment in clinically significant macular edema (CSME) by 50% [4]. Reported complications in patients with DME who undergo laser microaneurysm (MA) photocoagulation and/or grid photocoagulation include scotoma, visual field defects, and chorioretinal atrophy [5–7]. To address these risks, less invasive laser treatments have been proposed. In 1997, subthreshold micropulse photocoagulation was posited for the treatment of DME [8] and has since demonstrated efficacy in several studies [8–18]. Subthreshold micropulse photocoagulation is a minimally invasive form of laser photocoagulation in which no coagulation spot is observed; this treatment modality is useful for relieving edema while preserving macular function [10, 12, 15–19]. Alternatively, in 2005, Topcon developed a pattern scanning laser system capable of applying multiple laser spots in a short period of time. PASCAL streamline yellow (Topcon Medical Laser Systems, Santa Clara, CA, USA) with endpoint management (EPM) software permits grid pattern photocoagulation and the ability to calculate the level of subthreshold energy required [14, 19, 20]. Yet, few clinical studies have evaluated subthreshold photocoagulation using EPM with green-wavelength lasers [20]. Additionally, no prospective study has used a model of the same yellow-wavelength laser as that used in the present study. Therefore, the purpose of this study was to examine the safety and efficacy of subthreshold photocoagulation for DME using EPM and the PASCAL streamline yellow.

## 2. Methods

We conducted a prospective study between November 2013 and June 2014 with the approval of the ethics committee at St Luke's International Hospital in Tokyo, Japan. Outpatients with DME were recruited from the Retina Division of the Department of Ophthalmology using the following entry criteria: (1) a diagnosis of type 2 diabetes as per the World Health Organization (WHO) criteria [21] (men and women aged 20 years or older) with macular edema involving the fovea, active leakage observed on fluorescein angiography (FA), central macular thickness (CMT) ≥ 300 *μ*m as measured by optical coherence tomography (OCT) (Cirrus HD-OCT®, Carl Zeiss Meditec, Germany) (measured 3 times, signal strength ≥ 5), and best-corrected visual acuity (BCVA) of 0.3–1.0 logMAR (decimal visual acuity: 0.1–0.5); (2) nonturbid ocular media with favorable mydriasis and clear visualization of the fundus on imaging; (3) intraocular pressure < 21 mmHg (excluding patients taking prescription eye drops); (4) contraindication for direct microaneurysm photocoagulation lasting at least 12 weeks as determined by an ophthalmologist, availability to visit the hospital on examination days, and ability to comply with medical professional instructions; and (5) patient consent for necessary general ophthalmological examinations and OCT measurements. The exclusion criteria were as follows: (1) patients who underwent panretinal photocoagulation within the past month or patients requiring panretinal photocoagulation within the next 6 months; (2) an abnormal lesion in the area of the macular edema (including the deposition of hard exudate within the foveal avascular zone, the epiretinal membrane with retinal folds in the fovea, or chorioretinal atrophy in the macular area including the fovea) that is judged to interfere with the improvement in visual acuity; (3) ophthalmoscopically visible vitreomacular traction or that detected on OCT that is judged to cause macular edema; (4) macular edema caused by a disease other than diabetes; (5) atrophic scarring within 200 *μ*m of the foveal avascular zone due to past laser treatment; (6) atrophy, scarring, or subretinal fibrillization in the eye including the fovea; (7) patients who underwent yttrium-aluminum-garnet laser treatment, peripheral retinal photocoagulation (for a retinal tear), direct microaneurysm photocoagulation, or grid photocoagulation within the past 12 weeks; (8) decreased visual acuity, lack of a clear fundus image, or an unexaminable retinal lesion due to significantly turbid ocular media including cataract; (9) patients requiring cataract surgery within 1 year; (10) intraocular surgery within the past 6 months; (11) surgery for epiretinal membrane peeling or inner limiting membrane peeling; (12) surgery within the last month or cranial radiation therapy; (13) pharmacotherapy for DME including intravitreal, subconjunctival, or sub-Tenon's capsule injection of a corticosteroid within the last 90 days; (14) allergy to contrast dye for fluorescent fundus angiography; (15) acute eye infection or an infection around the eye; (16) congenital abnormality of the fundus including the optic nerve head; (17) severe renal dysfunction or hemodialysis; (18) glaucoma; and (19) patients judged to be inappropriate for study inclusion by a physician.

For patient screening, we measured visual acuity and intraocular pressure, obtained color photos of the fundus (TRC 50DX, Topcon Medical Laser Systems, Santa Clara, CA, USA), performed FA and fundus autofluorescence (FAF) imaging (Spectralis®, Heidelberg Engineering, Heidelberg, Germany), measured CMT and macular volume (MV) (Cirrus HD-OCT, Carl Zeiss Meditec, Germany), and measured macular sensitivity using microperimetry (Maia®, Topcon Medical Laser Systems, Santa Clara, CA, USA).

First, laser titillation was performed using life-size magnification lenses (Mainster Standard lens, 1.04x; and Area Centralis, 1.02x). The spot size was 200 *μ*m. The lowest level of light intensity generating a visible scar, which was determined with a single shot (size, 200 *μ*m; duration, 0.015 s) in titration mode, was considered to be the threshold. The energy of subthreshold photocoagulation was set to 50% of the calculated threshold with EPM and 0.5 spacing. The laser was used to irradiate a donut-shaped area of the macular area excluding the fovea (landmark on) using the circular macular grid pattern. The laser was also used to irradiate a 4 × 4 grid pattern (landmark on) outside of the donut-shaped area if necessary or a 2 × 2 grid pattern (landmark off) inside of the donut-shaped area when strong edema was observed near the fovea. We did not irradiate the area within 500 *μ*m of the center of the macula during initial treatment.

BCVA measurement, ophthalmoscopy (FAF), OCT, and microperimetry were performed at baseline, 1 week, 1 month, 3 months, and 6 months posttreatment. FA was performed at 3 and 6 months posttreatment.

SPSS software version 19.0 (IBM Corporation, Armonk, NY, USA) was used for all statistical analyses. The threshold for statistical significance was *P* < 0.05. The measurements and collation were examined by 2 retinal specialists with more than 7 years of experience with the interpretation of OCT images; the specialists were blinded to clinical findings. Since the present study was conducted prior to approval of intravitreous ranibizumab injection (IVR), we did not perform IVR as a rescue therapy.

## 3. Results

The study included 10 eyes from 10 consecutive patients (6 men and 4 women) who provided written informed consent for participation. The mean age was 64.6 ± 11.1 years. All treatments and examinations were completed in all 10 patients. Laser irradiation was performed once in 8 eyes (80%) and twice in 2 eyes (20%) based on the degree of improvement in DME (Table 1).

The patients' medical history included panretinal photocoagulation in all eyes more than 6 months prior to the study, direct microaneurysm photocoagulation in 2 eyes (20%) more than 12 weeks prior to the study, intravitreal injection of bevacizumab with approval by the ethics committee of St. Luke's International Hospital in 3 eyes (30%) more than 6 months prior to the study, grid photocoagulation in 7 eyes (70%) more than 12 weeks prior to the study, and posterior sub-Tenon's capsule injection of triamcinolone in 3 eyes (30%) more than 90 days prior to the study. Laser photocoagulation was performed using the circular pattern alone in 3 eyes (30%), circular +2 × 2 grid patterns in 5 eyes (50%), circular +4 × 4 grid patterns in 1 eye (10%), and circular +2 × 2 grid +4 × 4 grid patterns in 1 eye (10%).

**Figure 1:**
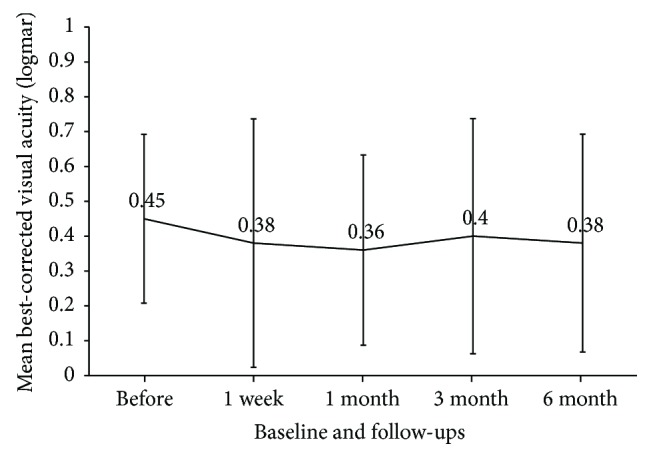
Mean best-corrected visual acuity (logMAR) at baseline and each follow-up.

There was no significant change in the mean BCVA (logMAR) value after treatment (Figure 1). Visual acuity was improved (0.2 logMAR) in 2 eyes (20%), unchanged in 7 eyes (70%), and exacerbated in 1 eye (10%) (Table 2).

**Figure 2:**
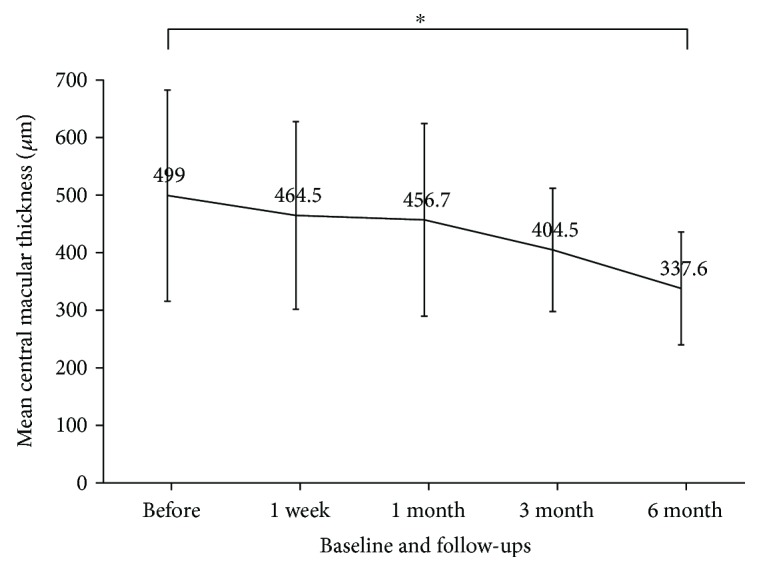
Mean central macular thickness (CMT) at baseline and each follow-up. Mean CMT was significantly decreased at 6 months posttreatment compared to baseline (Wilcoxon signed rank test, *P* = 0.024).

**Figure 3:**
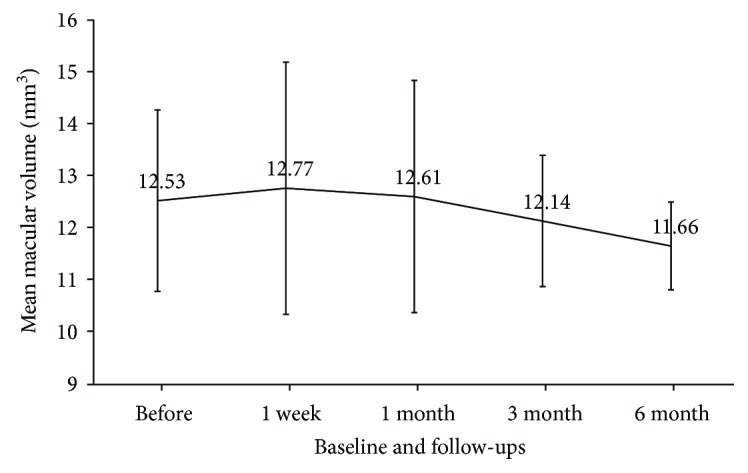
Mean macular volume at baseline and each follow-up.

Mean CMT was significantly decreased at 6 months posttreatment compared to baseline (499.0 *μ*m versus 337.6 *μ*m, respectively; Wilcoxon signed rank test, *P* = 0.024; Figure 2). Improvement by over 20% was observed in 5 eyes (50%), the CMT was unchanged in three eyes (30%), and exacerbation was observed in two eyes (20%; Table 2). Mean MV was not significantly changed after treatment (Figure 3). Improvement by over 10% was observed in two eyes (20%), and the MV was unchanged in 8 eyes (80%). No exacerbation of MV was observed (Table 2).

Spectral domain- (SD-) OCT examination before treatment revealed cystoid macular edema in all eyes. At 6 months posttreatment, edema was improved in 8 eyes (80%), unchanged in 1 eye (10%), and exacerbated in 1 eye (10%). Of 8 eyes demonstrating improvement after treatment, cystoid macular edema completely disappeared in 1 eye (12%; data not shown).

Three eyes (30%) had serous retinal detachment before treatment. At 6 months posttreatment, 2 eyes (67%) showed improvement and detachment was unchanged in 1 eye (33%). Serous retinal detachment completely disappeared in 1 (50%) of the 2 eyes showing improvement (data not shown).

FAF showed no change after treatment at the site of subthreshold photocoagulation in 4 eyes (40%), whereas there was an increase in FAF in punctate areas coinciding with coagulation spots in 6 eyes (60%). All 8 eyes (80%) showing a landmark showed increased autofluorescence. Fundus photographs showed no coagulation spots caused by subthreshold laser radiation at 6 months posttreatment (data not shown).

Imaging of the fundus prior to treatment revealed hard exudates near the macular area in 3 eyes (30%). At 6 months posttreatment, exudates had completely disappeared in two eyes (67%) and exacerbated in 1 eye (33%; data not shown). No subjective symptoms such as visual field defects or scotoma were observed.

**Figure 4:**
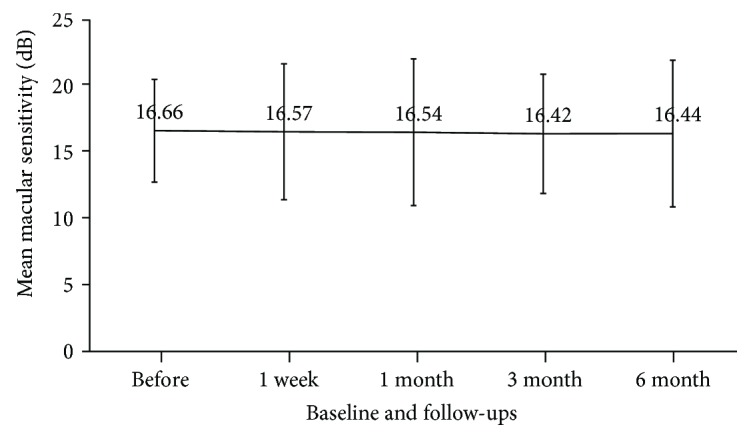
Mean macular sensitivity at baseline and each follow-up.

The mean retinal sensitivity in the macular area was not significantly changed after treatment (Figure 4). Although retinal sensitivity was improved by >1 dB in 4 eyes (40%) and exacerbated in 2 eyes (20%), these changes were not significant (Wilcoxon signed rank test, *P* = 0.46; Table 2).

All eyes showing decreased macular sensitivity after treatment exhibited an increase in autofluorescence coinciding with coagulation spots at 1 week posttreatment that was maintained through 6 months posttreatment. Macular edema on OCT completely disappeared in 4 eyes (40%), and macular sensitivity was increased in these eyes at 6 months posttreatment. FA findings showed hyperfluorescent spots in the macular area (microaneurysms) in the early period of angiography in 6 eyes (60%). Additionally, diffuse leakage of fluorescence was observed in the macular area during the middle and late periods in all eyes. Of the eyes showing hyperfluorescent spots, 5 (83%) showed improvement and 1 (17%) showed exacerbation at 6 months posttreatment (data not shown). With regard to diffuse leakage of fluorescence in the macular area during the late period of angiography, 8 eyes (80%) showed improvement, 1 eye (10%) showed no change, and 1 eye (10%) showed exacerbation at 6 months posttreatment (data not shown).

## 4. Case 1

**Figure 5:**
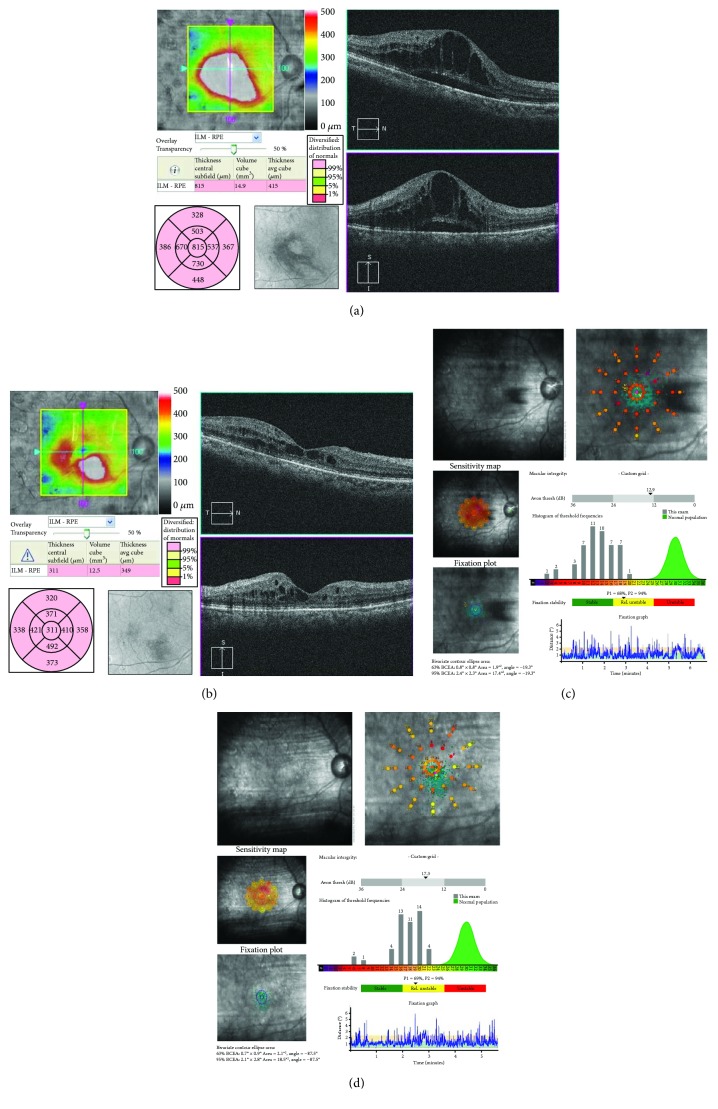
The right eye of a 70-year-old woman with diabetic macular edema. (a) Optical coherence tomography (OCT) before treatment. Macular edema containing cystoid macular edema and serous retinal detachment were observed in the fovea. (b) OCT at 6 months posttreatment. Cystoid macular edema was significantly reduced and serous retinal detachment completely disappeared. Additionally, foveal cupping appeared. (c) Macular sensitivity before treatment. (d) Macular sensitivity at 6 months after treatment. Sensitivity improved to 17.3 dB after treatment.

**Figure 6:**
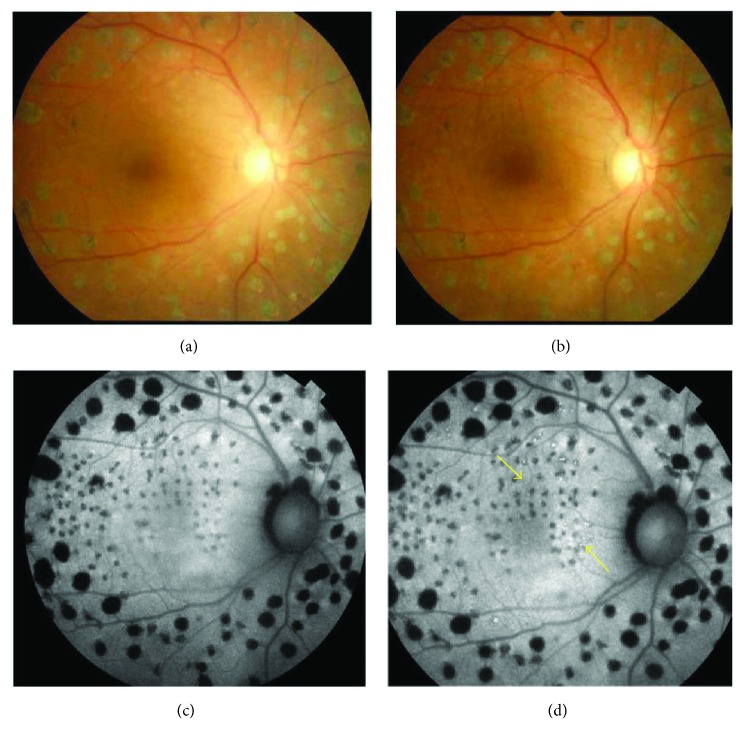
The right eye of a 70-year-old woman with diabetic macular edema (continued). (a) A color fundus photograph before treatment. Punctate retinal hemorrhages were observed around the macular area. (b) A color fundus photograph at 6 months after treatment. No laser scarring was observed in the macular area. (c) Fundus autofluorescence imaging before treatment. Hypofluorescent spots were observed at previous panretinal photocoagulation and grid photocoagulation sites. (d) Fundus autofluorescence imaging at 6 months after treatment. Autofluorescence was observed in the treated parafovea (yellow arrows).

A 70-year-old woman presented with DME of the right eye. The patient had undergone treatment once with minimally invasive grid photocoagulation, twice with posterior sub-Tenon's capsule injection of triamcinolone, and once with panretinal photocoagulation. Baseline measurements were as follows: BCVA (decimal visual acuity), 0.3; CMT, 815 *μ*m; MV, 14.9 mm^3^; and macular sensitivity (measured with the Maia), 12.9 dB. OCT revealed macular edema containing cystoid macular edema and serous retinal detachment in the fovea (Figure 5(a)). FAF showed hypofluorescent spots at previous panretinal photocoagulation and grid photocoagulation sites (Figure 6(c)). The conditions of laser photocoagulation were as follows: wavelength, 577 nm; spot size, 200 *μ*m; duration, 0.015 s; power, 250 mW; energy, 5.6 mJ (50%); and spacing, 0.5. A total of 169 laser shots were used to irradiate 4 spots (up, down, left, and right) in the circular +2 × 2 grid patterns. At 6 months after treatment, BCVA (decimal visual acuity) was 0.2 and MV was slightly decreased to 12.5 mm^3^ from baseline. Macular sensitivity in the center was 17.3 dB and demonstrated improvement after treatment (Figures 5(c) and 5(d)). FAF revealed autofluorescence around the macular area after treatment (Figures 6(c) and 6(d)). OCT showed a decrease in CMT from 815 *μ*m to 311 *μ*m, a significant reduction in cystoid macular edema, and the complete disappearance of serous retinal detachment. Additionally, foveal cupping appeared (Figures 5(a) and 5(b)).

## 5. Case 2

**Figure 7:**
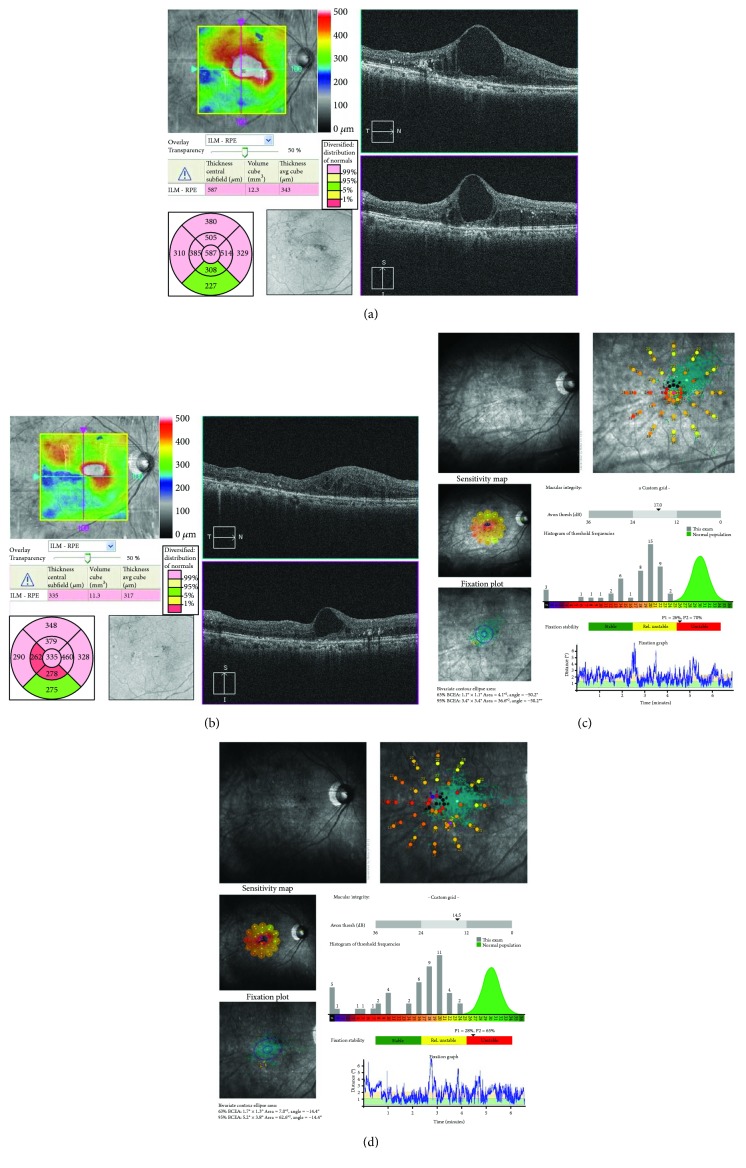
The right eye of a 75-year-old woman with diabetic macular edema. (a) Optical coherence tomography (OCT) before treatment. Diffuse macular edema with a cyst was observed in the fovea. (b) OCT at 6 months after treatment. The size of a giant cyst cavity in the fovea was significantly reduced and foveal cupping appeared. (c) Macular sensitivity before treatment. (d) Macular sensitivity after 6 months after treatment. Sensitivity improved to 14.5 dB after treatment.

**Figure 8:**
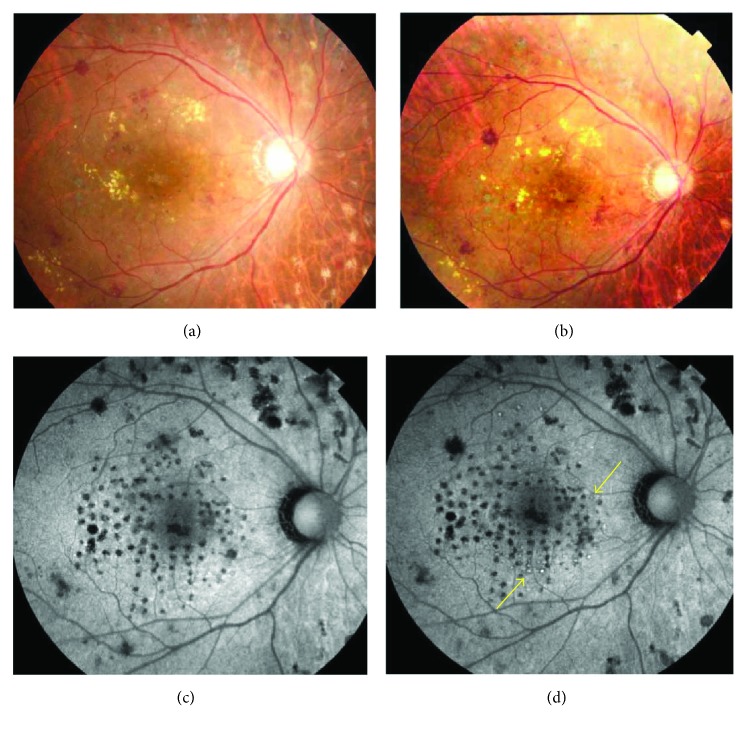
The right eye of a 75-year-old woman with diabetic macular edema (continued). (a) A color fundus photograph before treatment. Punctate retinal hemorrhages and hard exudates were observed around the macular area. (b) A color fundus photograph at six months after treatment. No laser scar was observed in the macular area. (c) Fundus autofluorescence (FAF) before treatment. Hypofluorescent spots were observed in the sites of panretinal photocoagulation and previous grid photocoagulation. (d) FAF at 6 months. Autofluorescence was observed in the parafovea (yellow arrows).

A 75-year-old woman presented with DME of the right eye. The patient had undergone treatment once with grid photocoagulation, twice with posterior sub-Tenon's capsule injection of triamcinolone, once with panretinal photocoagulation, and once with vitreous surgery. Baseline measurements were as follows: BCVA (decimal visual acuity), 0.1; CMT, 593 *μ*m; MV, 12.4 mm^3^; and macular sensitivity (measured with the Maia), 17.0 dB. OCT revealed diffuse macular edema with a cyst in the fovea. FAF showed hypofluorescent spots at previous panretinal photocoagulation and grid photocoagulation sites. The conditions of laser photocoagulation were as follows: wavelength, 577 nm; spot size, 200 *μ*m; duration, 0.015 s; power, 225 mW; energy, 4.5 mJ (50%); and spacing, 0.5. A total of 200 laser shots were used to irradiate the circular +4 × 4 grid patterns. At 6 months after treatment, BCVA (decimal visual acuity) was improved to 0.3. Both CMT (335 *μ*m) and MV (11.3 mm^3^) were lower after treatment than at baseline. Macular sensitivity in the center was 14.5 dB and demonstrated exacerbation after treatment (Figures 7(c) and 7(d)). FAF revealed autofluorescence in the parafovea (Figures 8(c) and 8(d)), and OCT showed that a giant cyst cavity in the fovea was significantly reduced, in addition to the appearance of foveal cupping (Figures 7(a) and 7(b)).

## 6. Discussion

In the present study, we performed subthreshold photocoagulation in 10 eyes from 10 patients with DME using EPM in the PASCAL system. The results showed that at 6 months posttreatment, CMT was significantly decreased, while there were no significant changes in macular sensitivity, mean BCVA (logMAR), or macular volume.

EPM is a software program installed in the PASCAL streamline yellow system that is used to quantify the degree of invasion with a unique algorithm [19, 20]. Subthreshold laser photocoagulation is a treatment modality in which coagulation spots are invisible due to the use of subthreshold energy (approximately 50% of the lowest level of energy that allows coagulation spots to be observed). For treatment, the degree of invasion into tissue varies depending on the energy used. When the amount of heat (power × exposure time in laser photocoagulation) is reduced, there is a corresponding nonlinear reduction in the degree of tissue invasion, regardless of whether exposure time or power is kept constant [19, 20]. In contrast, EPM quantifies the degree of invasion using the Arrhenius equation, which allows for the control of tissue invasion. In animal experiments, the energy setting of EPM was linearly correlated with the degree of invasion [19, 20]. EPM software enables an operator to perform photocoagulation for a given condition of invasion and thereby limit the invasiveness of photocoagulation.

At present, modified ETDRS laser treatment is a standard laser treatment for DME; however, modified ETDRS has been associated with damage in the outer and inner retinal layers, causing scotoma [4–7]. Alternatively, a short duration of irradiation (0.015 seconds) and subthreshold irradiation using EPM decreases the degree of invasion in animals. Lavinsky et al. [19] reported that irradiation at 50% of the EPM setting in rabbits allowed rapid recovery of the retinal pigment epithelium (RPE) and OCT findings showed that, while vertical highly reflective spots were observed in some areas of the RPE immediately after irradiation, these spots disappeared by 2 months posttreatment accompanied by reconstruction of the outer retinal layer [14]. Synapse reconstruction was also demonstrated over the same recovery period [19, 22]. Although we studied subthreshold photocoagulation in the human retina under different conditions in the present study, changes in FAF findings in some eyes may indicate that some invasion of the RPE occurred; however, the absence of changes in macular sensitivity suggests that the EPM is a method that does not largely affect macular function in the short or long term.

Our study showed that retinal sensitivity measured with the Maia was improved by 1 dB in 4 eyes (40%), unchanged in 4 eyes (40%), and exacerbated in 2 eyes (20%), although posttreatment changes were nonsignificant. In contrast, a study using micropulse laser treatment reported that retinal sensitivity was significantly improved after subthreshold grid photocoagulation, while sensitivity was decreased after conventional threshold grid laser treatment [10, 12, 18]. Hoshikawa et al. [12] reported that retinal sensitivity was improved in 38% of eyes and unchanged in 62% of eyes immediately after subthreshold photocoagulation using a micropulse laser. Our results are consistent with previous indications of the ability of subthreshold grid photocoagulation to improve retinal sensitivity compared to conventional laser treatment [8–10, 18].

The mechanism of successful subthreshold laser photocoagulation has been inferred in various studies. Inagaki et al. [13] showed that subthreshold photocoagulation of a monolayer culture of retinal pigment epithelial (ARPE-19) cells with a micropulse laser enhanced the expression of heat shock protein (Hsp). Hsp family members act as chaperone proteins to aid in refolding denatured proteins and protect against apoptosis and inflammation [23–27]. Hsp expression is enhanced by increases in temperature. In studies of ME, Hsp produces improvements without causing cell death. Upregulation of Hsp70 expression by laser irradiation is thought to play an important role in the improvement of macular edema [28]. Since FAF was changed in response to 50% EPM energy in our study, our protocol may have caused some mild damage to the RPE; in addition to increasing Hsp expression, reversible damage to the outer retinal layer may have improved oxygen delivery from the choroid to the inner layer [14, 19, 20, 22].

The present study had some limitations. First, this study was conducted at a single facility and included a small number of cases. Second, because this study was conducted prior to the clinical approval of IVR, it did not include IVR as a rescue treatment.

## 7. Conclusions

Although antivascular endothelial growth factor (VEGF) treatment remains the first-line treatment for DME, the use of adjunct laser treatment has been shown to reduce the number of injections necessary for patient management [29–43]. The results of future clinical studies are needed to corroborate our present findings and validate the utility of subthreshold photocoagulation. A combined treatment with less invasive laser treatment is expected to improve the safety and efficacy of clinical DME treatment.

## Figures and Tables

**Table 1:** Patient characteristics.

Study patient	Age	Sex	Follow-up (months)	Type of DM	Laser irradiation times
1	77	M	6	Type 2	1
2	66	F	6	Type 2	1
3	51	M	6	Type 2	1
4	70	F	6	Type 2	1
5	64	F	6	Type 2	1
6	75	F	6	Type 2	1
7	74	M	6	Type 2	2
8	41	M	6	Type 2	2
9	63	M	6	Type 2	1
10	65	M	6	Type 2	1

DM: diabetes mellitus.

**Table 2:** Improvements over the study follow-up period.

Examination	Improved (%)	Unchanged (%)	Exacerbated (%)
BCVA	2 (20)	7 (70)	1 (10)
CMT	5 (50)	3 (30)	2 (20)
MV	2 (20)	8 (80)	0 (0)
MS	4 (40)	4 (40)	2 (20)

BCVA: best-corrected visual acuity; CMT: central macular thickness; MS: macular sensitivity; MV: macular volume.
